# Mapping of Rice Varieties and Sowing Date Using X-Band SAR Data

**DOI:** 10.3390/s18010316

**Published:** 2018-01-22

**Authors:** Hoa Phan, Thuy Le Toan, Alexandre Bouvet, Lam Dao Nguyen, Tien Pham Duy, Mehrez Zribi

**Affiliations:** 1 Centre d’Etudes Spatiales de la Biosphère (CESBIO), 31401 Toulouse, France; thuy.letoan@cesbio.cnes.fr (T.L.T.); bouveta@cesbio.cnes.fr (A.B.); mehrez.zribi@ird.fr (M.Z.); 2 Telespazio France, 31023 Toulouse, France; 3 Space Technology Application Center, Ho Chi Minh City 700000, Vietnam; nlamdao@gmail.com; 4 An Giang University, An Giang 880000, Vietnam; pdtien@agu.edu.vn

**Keywords:** rice mapping, SAR, COSMO-SkyMed, rice variety, sowing date, Mekong Delta

## Abstract

Rice is a major staple food for nearly half of the world’s population and has a considerable contribution to the global agricultural economy. While spaceborne Synthetic Aperture Radar (SAR) data have proved to have great potential to provide rice cultivation area, few studies have been performed to provide practical information that meets the user requirements. In rice growing regions where the inter-field crop calendar is not uniform such as in the Mekong Delta in Vietnam, knowledge of the start of season on a field basis, along with the planted rice varieties, is very important for correct field management (timing of irrigation, fertilization, chemical treatment, harvest), and for market assessment of the rice production. The objective of this study is to develop methods using SAR data to retrieve in addition to the rice grown area, the sowing date, and the distinction between long and short cycle varieties. This study makes use of X-band SAR data from COSMO-SkyMed acquired from 19 August to 23 November 2013 covering the Chau Thanh and Thoai Son districts in An Giang province, Viet Nam, characterized by a complex cropping pattern. The SAR data have been analyzed as a function of rice parameters, and the temporal and polarization behaviors of the radar backscatter of different rice varieties have been interpreted physically. New backscatter indicators for the detection of rice paddy area, the estimation of the sowing date, and the mapping of the short cycle and long cycle rice varieties have been developed and assessed. Good accuracy has been found with 92% in rice grown area, 96% on rice long or short cycle, and a root mean square error of 4.3 days in sowing date. The results have been discussed regarding the generality of the methods with respect to the rice cultural practices and the SAR data characteristics.

## 1. Introduction

Rice plays an especially important economic role, being the staple food and the main source of livelihood of many people in agricultural producing countries such as Vietnam. Today the rice cultivated areas and rice production are being increasingly undermined by the effects of industrialization and climate change. Consequently, many farmers are changing their agronomic practices such as the use of new varieties, changes in water management, in fertilization, etc., which have impacts on the environment and also on international rice markets [1]. For instance, in the past 20 years, in order to increase the production, farmers have been turning to intensive cropping system with varieties of shorter cycle and higher yields. This requires the use of more chemical products (fertilizers and pesticides), leading to negative impacts on the environment. However, the short cycle rice usually has poor quality and a lower price in the global market. Recently, to increase their income, farmers and stakeholders of the rice markets have adopted high market value long cycle rice varieties, and low chemical input treatment. However, the cultivation practices and crop calendar are not uniform within a region. This leads to poor information on the market values of the rice production and on the timing of the crop growth stage, in particular sowing and harvest dates. Accurate information of rice production are therefore highly needed for governments, economic decision makers and farmers, who need to be informed about the spatial distribution of different rice varieties and of the key crop growth stages, for water management and crop treatment. 

To collect information on rice crops, remote sensing techniques have been developed and demonstrated using a large variety of optical and microwave remote sensing data in many studies [2,3]. The limitation of the frequent cloud cover over rice growing regions poses a challenge to the use of optical images during critical stages of the plant growth, in particular during the onset of the rainy season. Synthetic Aperture Radar (SAR) systems present an alternative for rice crop monitoring with two major advantages: their all-weather capability and the specific physical interaction between the radar waves and the rice canopy over inundated or moist soil surface. By far most studies have demonstrated the usefulness of SAR data at C, L and X band (e.g., ERS-1&2, RADARSAT-2, ENVISAT ASAR, ALOS PALSAR, COSMO-SkyMed, Sentinel-1, etc.) for mapping rice areas and for retrieving the rice biophysical characteristics, potentially leading to production forecasting [4,5,6,7,8,9,10,11,12,13]. A very common approach on delineating rice is based on the specific temporal variation of radar backscatter over the growing season: the backscattering intensity shows a significant increase during the vegetative phase, right after the low values of the flooding stage, and then decreases slightly during the reproductive phase until harvest. The increase was observed with X-band SAR in early investigations since 1990s from airborne SAR [14], and later on with ground-based X-band radar [15], spaceborne TerraSAR-X [16,17] and COSMO-SkyMed [18,19]. More works have been realized with C-band SAR using ERS, RADARSAT-1 or ASAR [20,21,22,23,24]. For example, the start of the season (SoS), which corresponds to the sowing or transplanting date, has been estimated in several studies, either as a mere step in the rice mapping process [20] or as a self-standing product [22]. This SoS is identified as the period during which the fields were flooded and therefore exhibited low $\sigma^{0}$. However, this assumption does not hold true everywhere, as modern agronomic practices (e.g., in Vietnam) consist in sowing rice on wet (not flooded) fields which have variable backscatter values due to the surface roughness. Other more reliable approaches need to be developed for these areas.

In this paper, we assess the use of X-band SAR data to retrieve the information on the sowing date and the rice varieties, those additional information of high interest in a region having modern and non-uniform cultivation practices. The work consists of an experiment carried out in the An Giang Province, Vietnam, where individual farmers have different planting schedules, growing practices and water management methods, which poses a great challenge for using Earth observation data. The study of the use of X-band SAR data for Vietnam is driven by the Vietnamese Satellite LOTUSat due to launch in 2020–2021. The SAR data from COSMO-SkyMed are analyzed as a function of the ground data, and their temporal variations are interpreted to derive indicators that can be used for rice mapping and for the retrieval of relevant rice information.

The paper is organized as follows: Section 2 will describe the test site and data used in this study, both the SAR data and ground data. The methodology will be presented in Section 3. The SAR data subsection includes image preprocessing and analysis as a function of ground data, to highlight growth phases of different rice varieties and then physical interpretation of the temporal and polarization behavior of the radar backscatter of the different rice varieties. Based on the analysis and interpretation results, methodologies are developed for the mapping of rice area, rice varieties, and field-scale sowing date. The results and discussions will be presented in Section 4. Section 5 will present the conclusions, with suggestions for future works.

## 2. Test Site and Data 

### 2.1. Test Site

**Figure 1:**
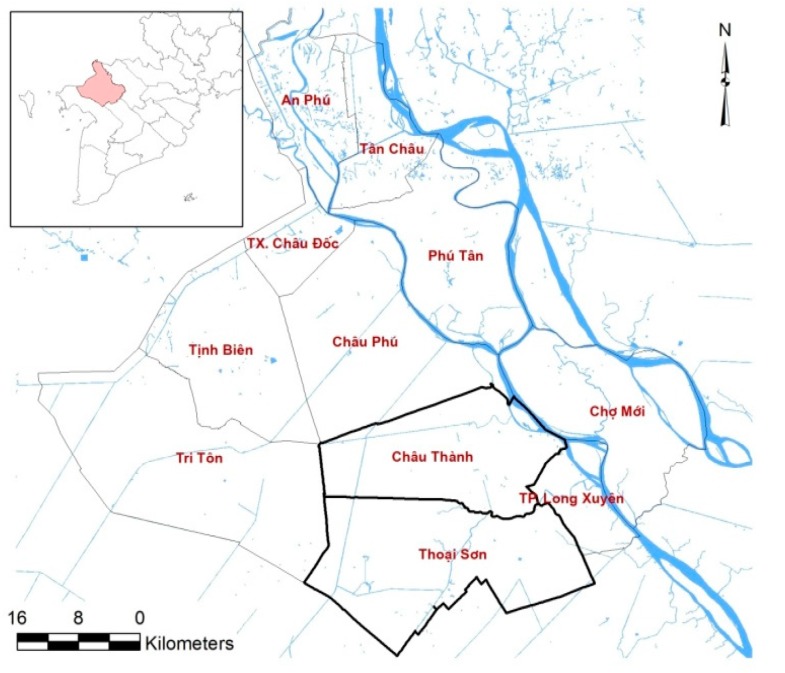
Location of An Giang Province and Chau Thanh, Thoai Son communes in the Mekong Delta Vietnam.

The test site is a part of the An Giang Province, located in the upper reach of the Mekong River Delta (MRD) in Vietnam, at the border with Cambodia, extending from 10°12′ to 10°57′ N latitude and 104°46′ to 105°35′ E longitude (Figure 1). An Giang has an area of about 3500 km^2^, with a population of about 2,231,000 inhabitants. The topography of the region is extremely flat, with most areas lying just above sea level (0.7–1.2 m in height). Agricultural land covers the largest area (278,800 ha), of which 85% (237,500 ha) are rice farms [25]. An Giang is the leading province of the country in terms of rice production, with more than two million tons produced yearly. 

The study area is composed of Chau Thanh and Thoai Son, two districts where rice growing areas occupy about 80% of the total area [26]. Chau Thanh and Thoai Son are pioneer areas promoting high-tech applications in agricultural development in An Giang Province. Rice farmers in the region have relatively small farms that are of 1 to 2 hectares on average and generally cultivate three major cropping seasons during a year: winter-spring or early season; summer-autumn or midseason; and autumn-winter or a longer rainy season crop, as shown in Table 1. Details about the rice cultivation practices of this region will be described in Section 3.1.1.

### 2.2. SAR Data 

**Figure 2:**
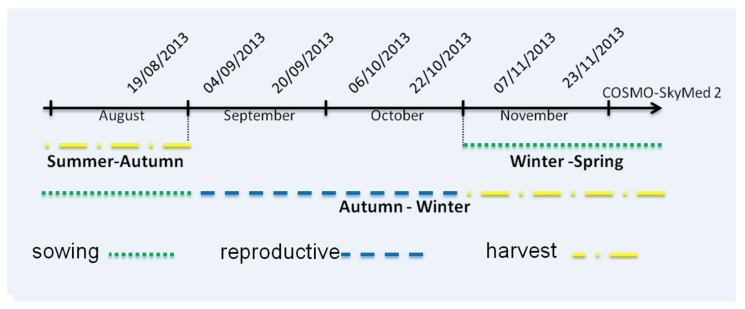
COSMO-SkyMed data available for the study and the rice crop calendar during this period in the An Giang Province.

The study uses COSMO-SkyMed (CSK) SAR data at X-band (9.6 GHz). COSMO-SkyMed is a constellation of four satellites, named CSK-1, CSK-2, CSK-3 and CSK-4. The data used were collected by CSK-2 with StripMap PingPong mode, of 17 km swathwidth at 10 m resolution, HH and VV polarizations and 16 days repeat cycle, at incidence angle of 45°–47°, and in descending mode. This study uses seven images covering the Chau Thanh and Thoai Son districts in An Giang Province from 19 August to 23 November 2013. Figure 2 shows the CSK data acquisition dates together with the calendar of the main rice crops during this period including the end of Summer-Autumn season in August, early stage of Winter-Spring season in November and most of the Autumn-Winter season (Table 1).

### 2.3. Ground Data

**Figure 3:**
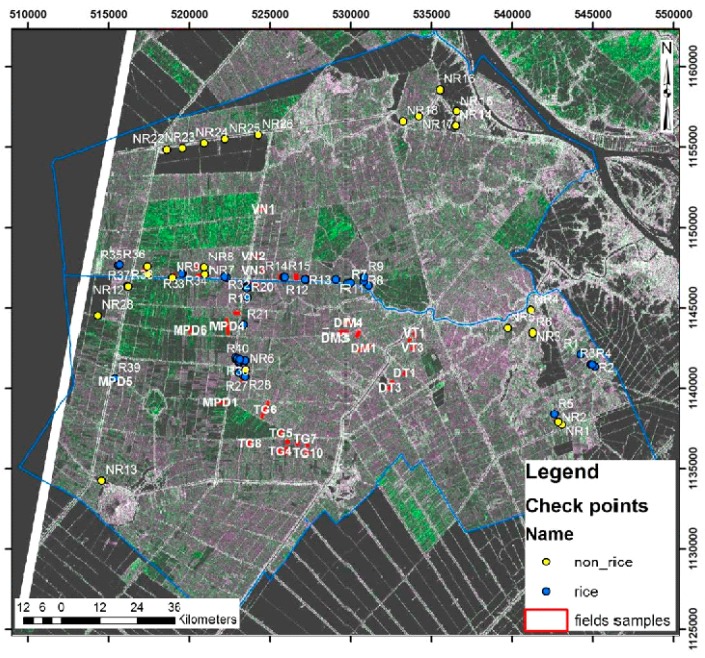
Forty rice field samples and 64 rice (blue points)/non-rice (yellow points) check points used for result validation. CSK image (magenta: HH, green: VV) of the Chau Thanh and Thoai Son districts, An Giang Province, 19 August 2013.

The collection of ground data is necessary to the understanding of the radar backscatter for algorithm development, and also for result validation. For algorithm development, forty rice fields located in Chau Thanh and Thoai Son districts were surveyed. To ensure low uncertainties in the mean radar backscatter over sampled fields, the fields were selected to have an area greater than 0.5 ha. For accuracy assessment of the rice mapping result, the rice growing acreage of 15 communes (the smaller administrative subdivision of a district) in Thoai Son district, obtained from the An Giang statistics office is used. Figure 3 shows the location of the 40 sampled rice fields and a number of non-rice check points in the Chau Thanh and Thoai Son districts, An Giang Province.

For 40 rice fields, ground data were collected nine times during the rice growth cycle in Autumn-Winter season. General parameters include rice varieties, date of sowing, planting methods (direct sowing, line sowing), date of harvesting, and final yield. Detailed parameters to be collected include plant height, water layer height, wet and dry biomass, Leaf Area Index (LAI), soil condition, etc. Note that not all the parameters were measured and collected in all fields for each of the survey dates, and in this study, the analysis will make use of the rice parameters pertaining to the detection of sowing date and the distinction of long and short cycle rice, which are rice variety, sowing and harvesting date, plant height. An example of ground data collected during SAR acquisition dates is shown in Table 2. The reference dataset of 40 rice sample fields can be found in supplementary files of the journal.

## 3. Methods 

The general methodology consists in three parts: (1) analysis and interpretation of in situ and SAR data, (2) derivation of SAR indicators for rice/non rice and rice varieties, and sowing date mapping, leading to (3) rice mapping methods.

### 3.1. Analysis and Interpretation 

#### 3.1.1. Ground Data Analysis

From the analysis of ground survey data from 40 rice fields, the following characteristics of rice fields in the region can be summarized as follows:

(1) Planting date and harvest date

Table 3 shows that the Autumn–Winter rice season in the region spanned from August 2013 to December 2013. The sowing dates for the same rice season can differ between the sampling fields by up to a month (from 02 August 2013 to 07 September 2013), although according to the common knowledge for Autumn–Winter crop sowing period in the region was mid-August. The harvest period spanned from 02 November 2013 to 17 December 2013. The growth duration of rice is from 80 to 110 days depending on the rice varieties. The cultivation practices and crop calendar are not uniform within the region, this leads to poor information on the market values of the rice production, and on the timing of the crop growth stage, in particular sowing and harvest dates. 

(2) Rice varieties

The dominant varieties grown were OM 4218 (42%), IR 50404 (39%), and jasmine (16%). Two groups of rice can be distinguished according to their growth cycle and yield. The short-grain rice variety IR 50404 has a short duration 80–86 days, a high and stable yield ranging from 6 to 8.5 ton/ha for the 15 sampled fields (average 7.6 t/ha). On the other hand, taking advantage of its short growth cycle, the farmers plant IR 50404 in the summer-autumn crop so they can still plant the autumn-winter crop before the flooding season begins in October-November. 

The long–grain, long growth cycle varieties include OM4218 (90–97 days) and Jasmine (95–110 days). The yield for 17 sampled fields of OM4218 is 4–7 ton/ha (average 6.5 ton/ha). The Jasmine variety has the longest duration and variable yield (4–8 ton/ha).

For rice production, it appears more advantageous to plant short-cycle rice varieties. However, the market price is much higher for long-cycle rice, because of the rice grain quality. As an example, the market price in An Giang in March 2014 was of 263 USD a ton for IR 50404 and 417 USD a ton for jasmine rice [27]. As a consequence, the long-cycle rice, long time abandoned, is more and more used in the region. 

(3) Plant height 

An analysis of the height of rice plants as a function of sowing date shows a steadily increase until 70 days for short cycle rice and 85 days for long cycle rice. This trend has been observed in previous studies monitoring paddy rice fields [28]. The analysis is applied separately to nine samples of long-cycle rice fields (jasmine and OM4218) and nine samples of short-cycle rice fields (50,404), among the 40 sample fields. These 18 samples have been chosen to have close sowing dates, the same planting practices in order to avoid the effect of weather conditions in the trend analysis. 

**Figure 4:**
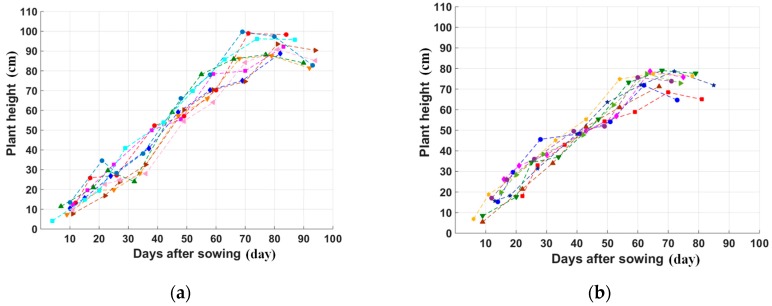
Temporal variation of plant height (versus days after sowing): (**a**) 9 sampling fields for long-cycle rice plants; (**b**) 9 sampling fields for short-cycle rice plants. The fields have been chosen to have close sowing dates and same planting practices.

Figure 4a shows the temporal variation of the plant height on nine samples in the My Phu Nhuan and Thoai Giang communes in Thoai Son district. The variation was observed on the same long-cycle varieties. The general increasing trend is observed until 80 days at 90–100 cm (reproductive stage) and is followed by a slow decrease. In the other Dinh My and My Phu Dong communes, nine samples of the same short-cycle variety show the increasing trend until 70 days at 80 cm maximum, followed by a plateau or a slight decrease as shown in Figure 4b. 

(4) Plant phenological stages

**Figure 5:**
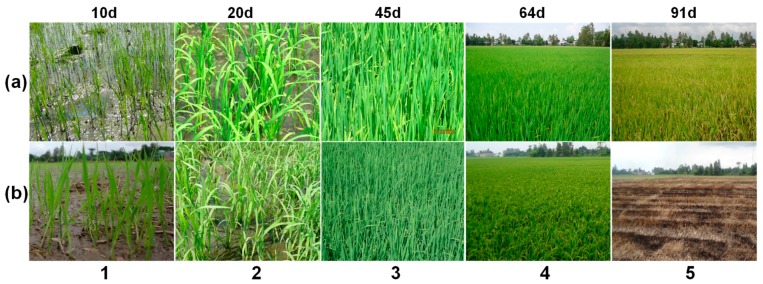
Ground survey photos at 10, 20, 45, 64, 91 days after sowing: (**a**) Long cycle rice variety, (**b**) Short cycle rice variety.

Figure 5 shows photographs of the rice fields at different growth stages, for two rice varieties: (a) long-cycle rice, (b) short-cycle rice. 

For each rice variety, the rice plants exhibit a vertical structure at the following stages: at the vegetative phase before tillering, and at the reproductive phase before maturation phase. This corresponds to Figure 5a(1,3, and 4), and to Figure 5b(1,3). The plants lose the vertical structure at 20 days (beginning of tillering), as can be seen in Figure 5a(2),b(2), and at maturity phase as can be seen in Figure 5a(5),b(4).

Figure 5 also shows that the difference between long-cycle and short-cycle rice varieties resides in the length of reproductive phase. The differences can be observed in Figure 5a(5),b(5).

#### 3.1.2. SAR Data Analysis and Interpretation

(a) SAR data pre-processing

In the first step, the CSK-2 level 1 images are preprocessed for the following:

❖ Calibration: conversion to the radar backscattering coefficient sigma nought ($\sigma^{0}$) from the digital numbers, which follows the procedure specified by the European Space Agency (ESA) [29];
❖ Geo-correction: Due to the topographical variations of a scene and the tilt of satellite sensor, distances can be distorted in the SAR images. Range-Doppler Terrain Correction is used to compensate for these distortions so that the geometric representation of the image will be corrected; these two operations are performed using the Sentinel Application Platform (SNAP) (http://step.esa.int/main/toolboxes/snap/).
❖ Filtering: All the algorithms developed in this study require a prior reduction of the speckle noise in the SAR images. One effective approach to reduce the speckle effect while preserving the spatial resolution is to apply a multi-image speckle filter [30,31] (multi-temporal and double polarization images in this study). The filter linearly combines M input images on a pixel-to-pixel basis, to create M output images with reduced speckle and thus increase the original number of looks L in the image to a higher equivalent number of looks (ENL), without reducing the spatial resolution. This filter requires the estimation of local averages in a spatial window of N pixels. It is shown in [31] that the ENL can be expressed as:
  $$\mathrm{ENL}=\frac{M\times N\times L}{M+N-1}$$

The size of the spatial window can therefore be chosen to meet a required ENL value. The required ENL can be assessed in order to meet a given probability of error in the rice/non-rice classification problem, as will be described in Section 3.2.2.

(b) The temporal radar backscattering of rice fields

**Figure 6:**
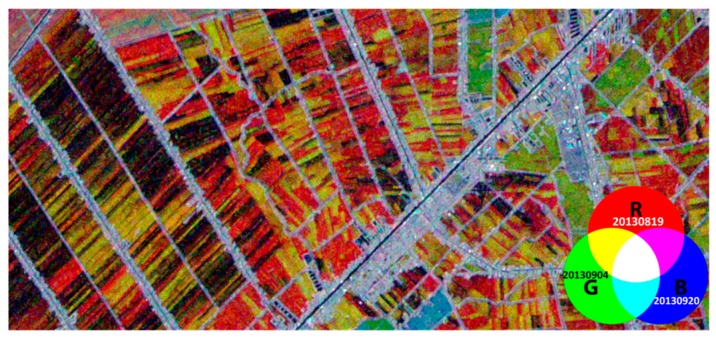
Example of RGB combinations of different dates (R: 19/08/2013, G: 04/09/2013, B: 20/09/2013) from CSK images, HH polarization over rice fields in the An Giang Province.

For a given date, the inter-field variation of the backscattering coefficient of 40 fields can be very large, up to 20 dB, notably at the beginning of the season, 19 August 2013 and 4 September 2013 and at the end of the season 23 November 2013, where the fields had a diversity of status, from bare fields, flooded or not, to rice plants at early and late growth stages. This backscatter variation reflects the inter-field variation of the crop calendar in the An Giang Province (and in the Mekong Delta in general), as described in the previous section, in terms of sowing date and the duration of the growth cycle. This is illustrated in the example in Figure 6. 

Figure 6 shows an example of RGB combinations of three dates from CSK images over rice fields in the An Giang Province using the HH polarization. It is to be noted that the crop type in the region is constituted almost entirely by rice (98%).

To interpret the backscatter temporal variation of individual rice fields, the backscatter coefficients need to be expressed as a function of the plant age (days after sowing). 

**Figure 7:**
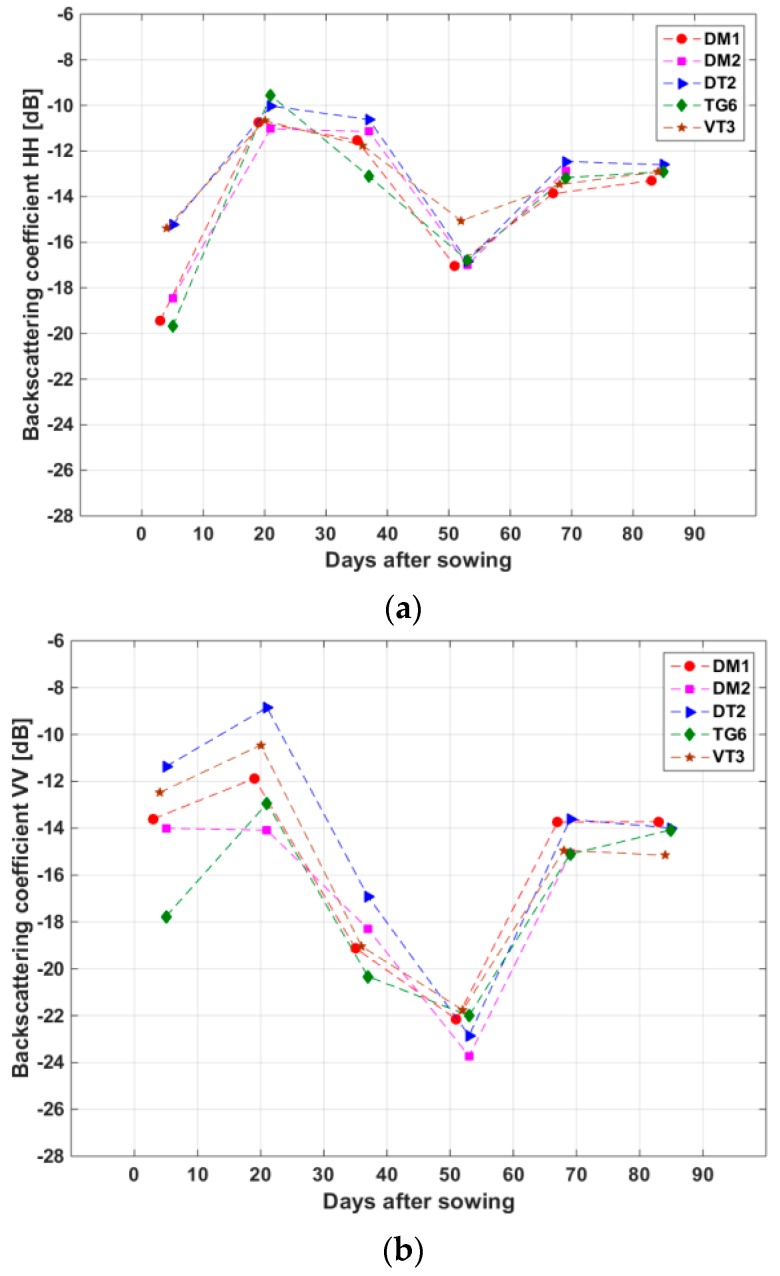

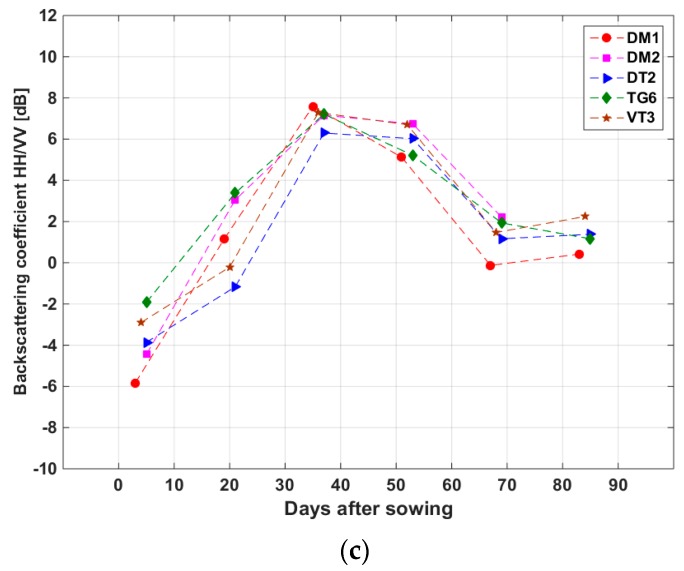
Variation of (**a**) HH, (**b**) VV backscattering coefficient, and (**c**) HH/VV of the five selected short-cycle sampled fields extracted from CSK images of six dates, versus the sowing date of each field.

In order to interpret physically the backscatter temporal variations of the rice canopy, we have analysed the backscatter of fields selected in such a way that the effects of cycle duration, meteorological conditions, and planting practices are minimised. For example, Figure 7 shows the backscatter of five fields planted with the same short cycle rice (variety 50404), close sowing dates (14–16 August), having the same method of direct sowing, from 3 to 5 days after sowing until harvest shortly after the last date. The temporal trend is similar among the 5 fields for each polarization, but as seen previously, the trend differs significantly between polarizations. The observations are in good agreement with the theoretical modelling of rice backscatter at X-band in Le Toan et al. [14]:

- From sowing to the beginning of tillering (0 to 20 days, plant height 0 to 20 cm, water in the fields from 10 to 20 days), an increase is observed at VV and HH, because of the increase in volume and double bounce scattering with the plant growth, the latter being largely dominant. At the first date, the fields were mostly wet bare soil. The backscatter has low backscatter values with large dispersion (5 to 6 dB), which can be interpreted as the effect of inter-field differences in soil moisture and surface roughness. From 0 to 20 days, VV is higher than HH, which is interpreted which was found in Le Toan et al., [14] as due to the double bounce scattering from vertical plant elements, the attenuation by these low biomass elements being negligible. At 20 days, when the plants lose their vertical structure, HH and VV are observed to have close backscatter values, with VV having more dispersion due to increasing attenuation by the plants. The resulting polarization ratio HH/VV exhibits also an increase from −4 ± 2 dB to 0.5 ± 2 dB.
- From the tillering to the beginning of the reproductive phase (20 to 35 days, plant height from 20 cm to 40 cm), the double bounce scattering decreases. At the same time, with the increase of biomass in the vertical stem, the plant attenuation increases, and in addition, the double bounce scattering, generates more backscatter in HH than in VV [32,33] leading to a much stronger decrease (7 to 8 dB) in VV, than in HH (1 to 2 dB). The resulting polarization ratio HH/VV increases up to 6 dB.
- From the beginning of the reproductive phase to the flowering stage (35 to 55 days, height from 40 cm to 60 cm, wet biomass from 2.5 to 4.5 kg/m², water in the fields between 40 to 50 days), the canopy was dense, leading to wave attenuation in both VV and HH. VV backscatter reaches a minimum value of −22 to −24 dB, HH reaching −15 to −17 dB. The resulting polarization ratio decreases slightly around 6 dB.
- From flowering stage to grain maturity phase (55 to 70 days, height from 60 cm to 80 cm), the attenuation by the stem is similar to that of the previous period. However, the biomass of the upper part of the plant increases. This results in an important increase of VV (8 dB) and of HH (4 dB). The resulting polarization ratio decreases to 0 to 2 dB.

From grain maturity to harvest (70 to 85 days, height from 80 to 65 cm, no water in the fields), there is small change in both HH and VV. The polarization ratio increases slightly from 0 to 2 dB.

It is interesting to note that the temporal variation of HH/VV follows well the plant growth, similarly to optical indicator such as Normalized Difference Vegetation Index (NDVI), confirming previous findings [34,35,36]. This is because the ratio reduces the effect of ground surface in the backscatter, maximizing the sensitivity to plant growth. 

To compare the temporal backscatter trajectory of the long-cycle rice, five fields of the same long-cycle variety, with the same method of sowing, and with a sowing date close to that of the five short-cycle rice fields of Figure 7 have been selected. 

**Figure 8:**
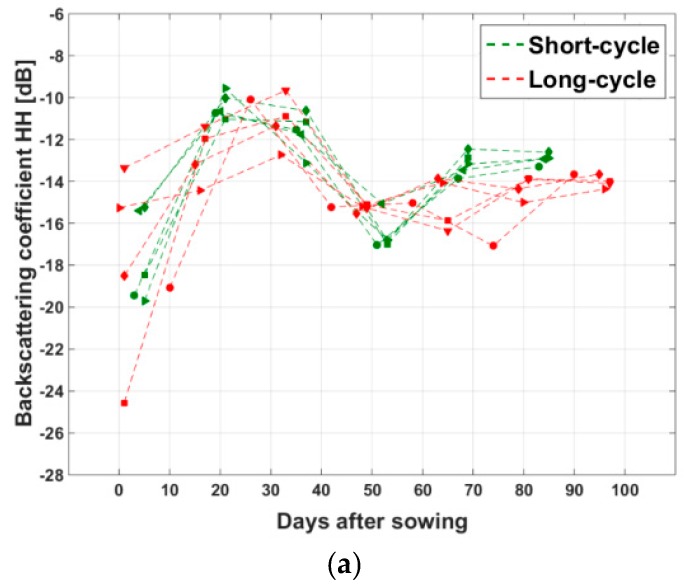

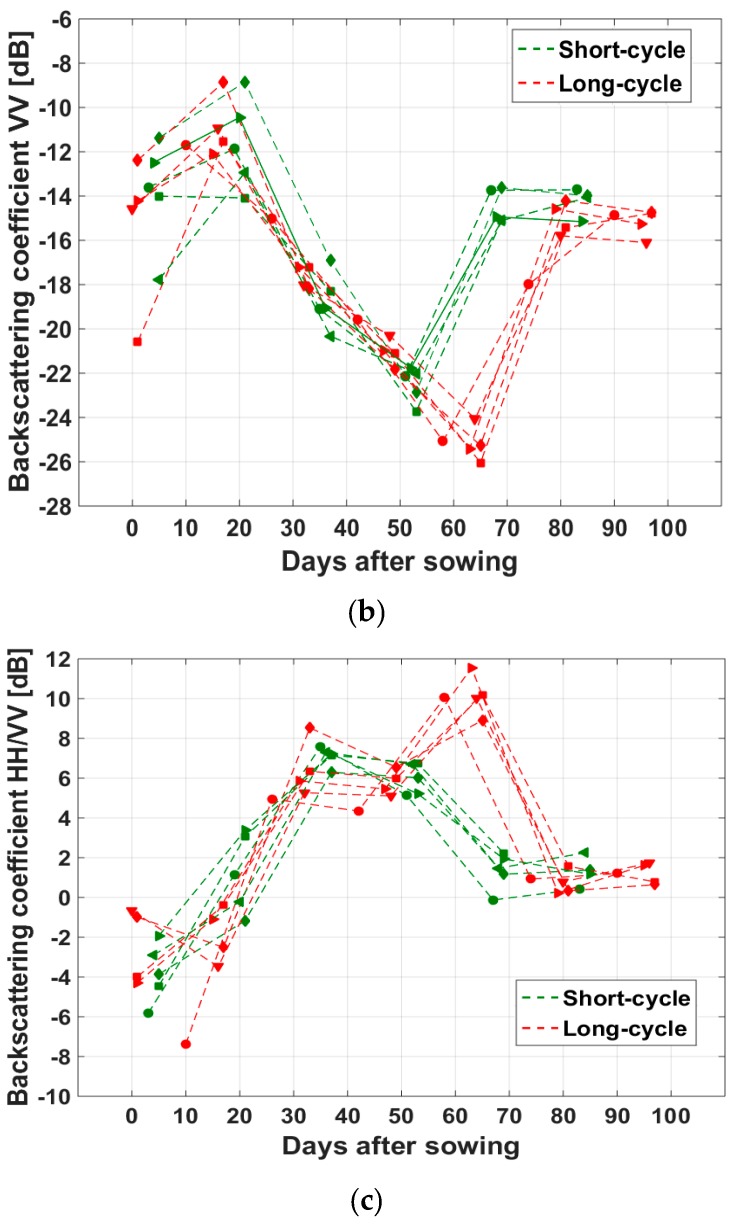
Variation of (**a**) HH, (**b**) VV backscattering coefficient, and (**c**) HH/VV of the 5 selected short -cycle sampled fields (in green), and 5 long-cycle fields (in red) extracted from CSK images of 6 dates, versus the sowing date of each field.

Figure 8 shows the backscatter variations of these selected long-cycle rice fields, as compared to the previous short-cycle fields. Similar trends in HH, VV, and HH/VV are observed with the two types of rice fields until 50–55 days after sowing. The latter period corresponds to the flowering stage of the short-cycle rice. After 55 days, the VV backscatter of short-cycle rice increases, whereas the VV of long-cycle rice continues to decrease until a minimum at 65 days, which corresponds to the flowering stage. On the contrary, HH does not show a clear backscatter trend after 50 days. The resulting HH/VV exhibits an increase until 35 days, followed by a plateau until 50 days, then a second peak appears at 65 days, followed by a strong decrease until 80 days, for which the two rice types have values in the range of 0 to 2 dB. Finally from 80 to 100 days, the HH/VV of the long-cycle rice remains stable. 

In addition, to generate the rice map, the radar backscatter of CSK images of other land use land cover (LULC) classes was analyzed. As previously stated, the study area is composed of Chau Thanh and Thoai Son, two districts where the crop areas account for 81.35%. The other LULC are water, aquaculture, forest, urban area. Among the crops, rice accounts for 98.34%, the other crops which are sugarcane, maize, vegetables occupy only 1.66%, quite negligible.

**Figure 9:**
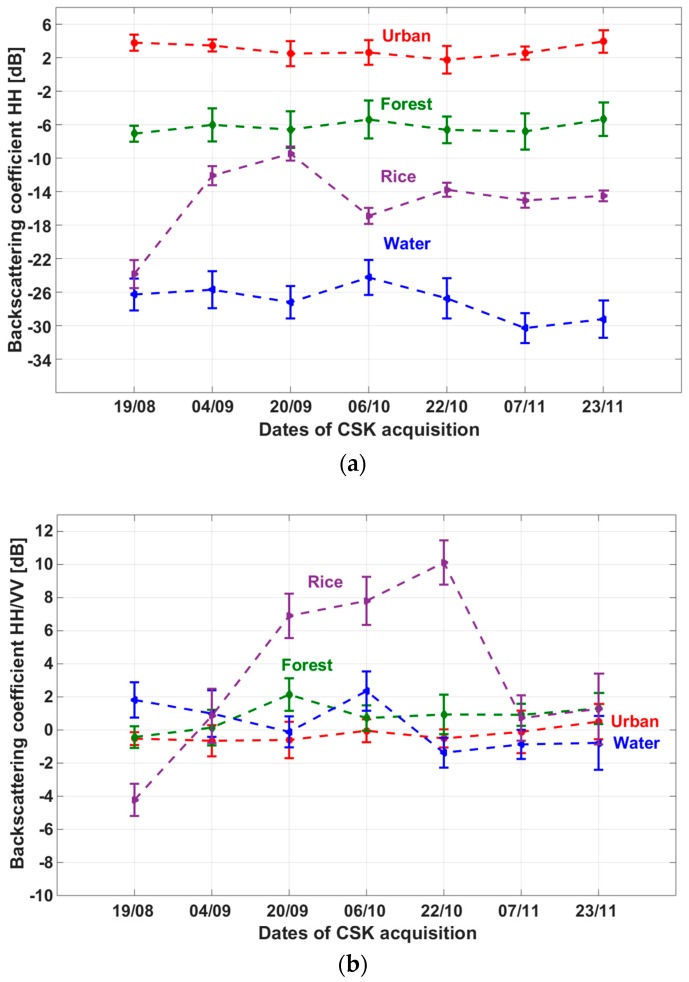
Temporal variation and standard deviation of (**a**) HH backscattering coefficient and (**b**) polarization ratio HH/VV of the four LULC sampled classes: forest in green, urban in red, river (water) in blue and rice in violet, extracted from CSK images of seven dates.

The backscatter signatures and their standard deviations of HH and the ratio HH/VV as a function of time from 19 August 2013 to 23 November 2013 are shown in Figure 9a,b of a rice field together with three other land cover types in An Giang Province: forest, urban, and river. The training samples (polygons) were homogeneous: each of them was made of 40 to 50 pixels of the same land cover type. The standard deviations were found about 1 to 2 dB. It is clearly seen that the forest, urban and river classes all exhibit lower temporal variability (less than 4 dB) than rice (up to 14 dB) on either HH or ratio HH/VV. The temporal changes of the backscatter and backscatter ratio are therefore strong indicators to separate rice from other land cover types. This feature can therefore be exploited for rice mapping. In addition, Figure 9a also indicates that it is possible to use the backscatter values at one polarization (HH) to separate the main land cover classes. Urban or built up area class has the highest backscatter signature (2 ± 2 dB). High backscatter also occurs for forest class ranging around −6 ± 2 dB. In contrast, the lowest backscatter is observed with water surface (lower than −22 dB) because of specular reflection (both for water bodies and flooded bare fields).

Figure 9b also illustrates the impacts of the vertical rice structure on the HH/VV ratio. As the plants grows, the vertical structure of the plants between tillering and maturity phase (shown in Figure 5) explains that HH is higher than VV because of the attenuation of the wave by the vertical stems. Compared to other LULC classes, rice fields are characterized by HH/VV outside the range of −3 dB, +3 dB during a great part of the growth cycle. It is to be noted that the fields of other crop (sugarcane, maize and vegetables) are quite small and not located in the in situ data base, so that they could not be analysed. However, in the study by Le Toan et al., [14], it was found that at X-band, many crop types such as wheat, sunflower, soybean have HH close to VV, even their backscatter values vary with time.

### 3.2. Rice Monitoring Methodology

#### 3.2.1. Mapping Indicators 

From the analysis results, we can derive several indicators for rice mapping, mapping of short-cycle and-long cycle rice, and determination of the sowing date for each rice field:

(a) Rice mapping: to discriminate rice fields from other LULC the temporal change of the HH and HH/VV ratio can be used. However, if the data acquisitions are not frequent, the data at the beginning or the end of a given rice season can be missed, polarization ratio HH/VV is a better indicator than HH for rice field mapping. In addition, indicators derived from the HH polarization (e.g., the maximum and minimum backscatter values in the HH time series) can be used to map other land use land cover classes (water, forest, and built up area), which can be used to reduce confusion in the rice map.
(b) Mapping of short/long cycle rice: in Figure 8c, the HH/VV ratio of the long cycle rice peaks at around 60 days after sowing at values of more than 10 dB (as compared to less than 8 dB at around 50 days after sowing for the short cycle rice). This was explained by the vertical structure of the plants (shown in Figure 5a(4)), and the plant height increasing from 80 cm to 100 cm, at 60 days to 80 days. The indicator to be used is therefore the maximum values of HH/VV.
(c) Determination of sowing date: as seen in Figure 7c and Figure 8c, the data dispersion in the HH/VV temporal profiles is small at the beginning of the season, where short-cycle and long-cycle varieties follow a similar development. In particular, it is observed over the 40 monitored fields that the HH/VV temporal curves cross the 0 dB line at 19.6 ± 4.3 days after sowing. This can be physically explained by the fact that this date corresponds to the beginning of tillering, when there is a drastic change in the plant structure: the leaves lose their vertical orientation (Figure 5a(2),b(2)), this feature, which was already observed in a previous study [14], can be used for the retrieval of the sowing date. However, the number of days after sowing at which HH/VV crosses 0 dB may depend on other factors such as cultural practice (direct sowing or transplanting) and the incidence angle. In the study by Inoue et al., 2002 [37], HH/VV ratio around 0 dB can be reached around 10–20 days, with a dependence on the incidence angle by up to 10 days. As a consequence, the method should require recalibration to be transferred to other sites/data.

#### 3.2.2. Methodology

**Figure 10:**
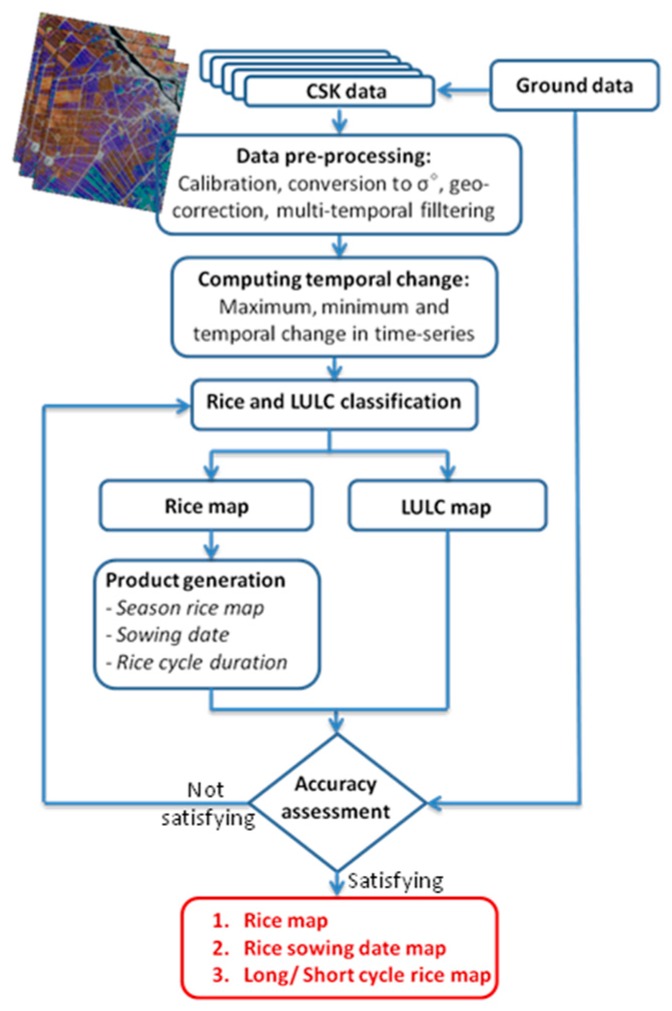
Flowchart of the rice monitoring method using multi-temporal CSK images.

Figure 10 shows the flowchart for rice monitoring using CSK data.

The preprocessing step includes calibration, registration, geo-correction, multi-temporal filtering as described in Section 3.1.2. Statistical features (maximum, minimum, temporal change) of the backscatter are computed for each pixel over the time-series by using Matlab. The maximum value of the polarization ratio HH/VV ($Max\_\sigma_{HH/VV}$) over the time-series will be used for rice mapping using the indicator (1):

(1) $$\begin{array}{l} \mathrm{If}\;\mathrm{Max}\_\sigma_{\mathrm{HH}/\mathrm{VV}}\;\geq \;T_{1}\;\mathrm{dB}\;\mathrm{then}\;\mathrm{Rice}\\ \mathrm{Else}\;\mathrm{If}\;\mathrm{Min}\_\sigma_{\mathrm{HH}}<T_{2}\;\mathrm{dB}\;\mathrm{then}\;\mathrm{Water}\\ \mathrm{Else}\;\mathrm{If}\;\mathrm{Max}\_\sigma_{\mathrm{HH}}>T_{3}\;\mathrm{dB}\;\mathrm{then}\;\mathrm{Built}\;\mathrm{areas}\;\mathrm{and}\;\mathrm{Trees} \end{array}$$

The threshold should be optimized considering the number of data acquisitions and other constraints such as local crop calendar, land use change, etc. [9]. To define the thresholds used in (1) the backscatter values of a high number of samples of rice, water, trees, urban, areas were selected by visual interpretation of Google Earth images, and for rice we retain the thresholds that minimize the error calculated using the 40 rice reference samples. In this study, a threshold of $T_{1}$ = 4 dB was applied for rice/non-rice classification. For this rice mapping method, SAR images from the beginning of the reproductive phase (35 days after sowing) are required. This rice/non-rice classification threshold can be used to estimate the required ENL following the approach described in Bouvet et al. [38]. The probability of error (PE) of the classification depends on the ENL and on Δr, the difference between the average polarization ratio HH/VV of the two classes (rice and non-rice). It can be seen in Figure 9 that the maximum HH/VV is always higher than 8 dB for the rice class and lower than 4 dB for the non-rice class. Δr is therefore higher than 4 dB. For this value of Δr, the theoretical expression of PE given in [38] indicates that an ENL value higher than 25 is required to guarantee a probability of error lower than 5%. In this study based on 14 CSK images (7 dates and two polarizations) with an original number of looks L equal to 2.88, the minimum number of pixels N of the spatial window of the multi-image filter is equal to 22 according to Equation (1). A 5 × 5 square window (25 pixels) was therefore used for the multi-temporal filter, resulting in an ENL of 26.5.

In terms of other LULC pixels, indicators for general land use land cover mapping are applied by the following rules using HH polarization: (a) the backscatter of water, being consistently low though slightly variable, a threshold of $\min\_\sigma_{\mathrm{HH}}<T_{2}\;dB$ was applied to map the water class; (b) the backscatter of urban areas and trees/forests having a consistently high backscatter compared to the other land use types in this area, a threshold of $\max\_\sigma_{\mathrm{HH}}>T_{3}\;dB$ was applied to map the built areas and trees. Other pixels not selected by those rules will be classified as other LULC class which can be other type of crops (sugar cane, corn, vegetable) and natural vegetation (grass, bushes). The methodology was applied in this study using $T_{2}$= −24 dB, $T_{3}$= −10 dB. 

For sowing date mapping, the method was based on the temporal behavior of the HH/VV value of rice pixels as described in Section 3.2.1:

(2) $$\mathrm{If}\;\mathrm{Date}\;=\;\mathrm{Date}\;(\sigma_{\mathrm{HH}/\mathrm{VV}}=0)\;\mathrm{then}\;\mathrm{Sowing}\;\mathrm{date}\;=\;\mathrm{Date}\;-20$$

The sowing date was estimated for each pixel by interpolating the HH/VV temporal curve and defining the plant age as 20 days before the date when the HH/VV curve crosses the 0 dB value. This method requires at least 3 images during the first part of the season (for example 4, 20, 36 days after sowing for the 16 days repeat cycle of CSK). 

The algorithm of short/long rice mapping was based on maximum value of the polarization ratio HH/VV. In this study, the threshold of the maximum polarization ratio of 8 dB was used to distinguish long and short duration cycle rice varieties:

(3) $$\begin{array}{l} \mathrm{If}\;\mathrm{Max}\_\sigma_{\mathrm{HH}/\mathrm{VV}}\;>8\;\mathrm{dB}\;\mathrm{then}\;\mathrm{long}\;\mathrm{duration}\;\mathrm{cycle}\;\mathrm{rice}\\ \mathrm{Else}\;\mathrm{If}\;\mathrm{Max}\_\sigma_{\mathrm{HH}/\mathrm{VV}}\leq 8\;\mathrm{dB}\;\mathrm{then}\;\mathrm{short}\;\mathrm{duration}\;\mathrm{cycle}\;\mathrm{rice} \end{array}$$

This method for discrimination of long/short cycle rice requires the knowledge of sowing date obtained by the method developed in the previous section, and the results are obtained only during the reproductive phase, from flowering stage to grain maturity phase. For this purpose, SAR images at around 60 days after the sowing date are required.

## 4. Results and Discussions

### 4.1. Rice Field Mapping

**Figure 11:**
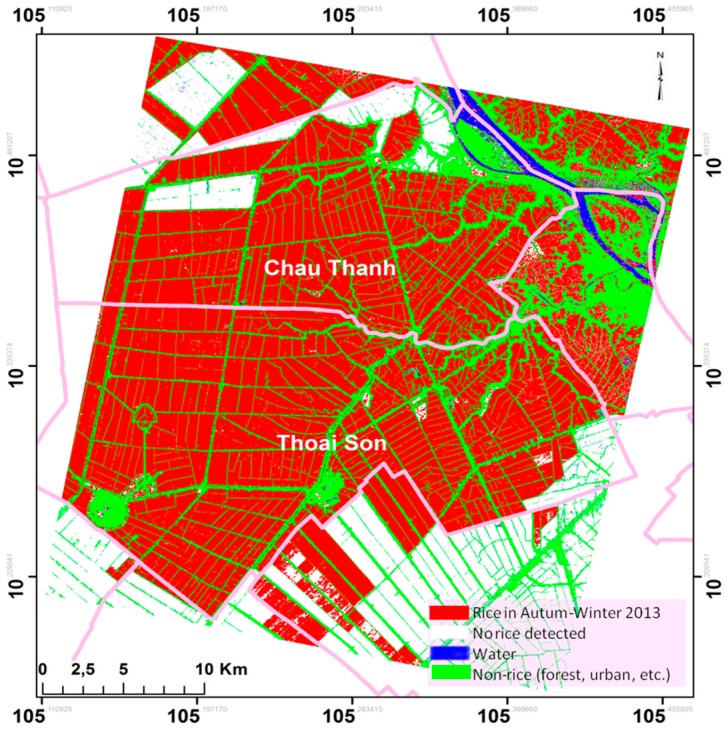
Rice and non-rice map of Autumn-Winter season in 2013. Red: Rice at Autumn-Winter; Blue: Water; Green: built areas and trees; White: no rice detected.

The rice mapping has been produced for Autumn-Winter rice season in 2013 in the CSK frame covering Chau Thanh and Thoai son district, in the An Giang Province. The resulting map in Figure 11 has been obtained by applying a 4 dB threshold on the maximum polarization ratio HH/VV in the time series (seven CSK images). The final rice/non-rice map is a combination of rice map and non-rice map (water, built areas and trees/forests, non-rice in Autumn-Winter rice season) generated according to the method previously described. The final result is obtained after post processing enhancement of the map, such as filtering of individual outlier pixels (for example using medfilt2 from the Matlab software).

### 4.2. Sowing Date Mapping

**Figure 12:**
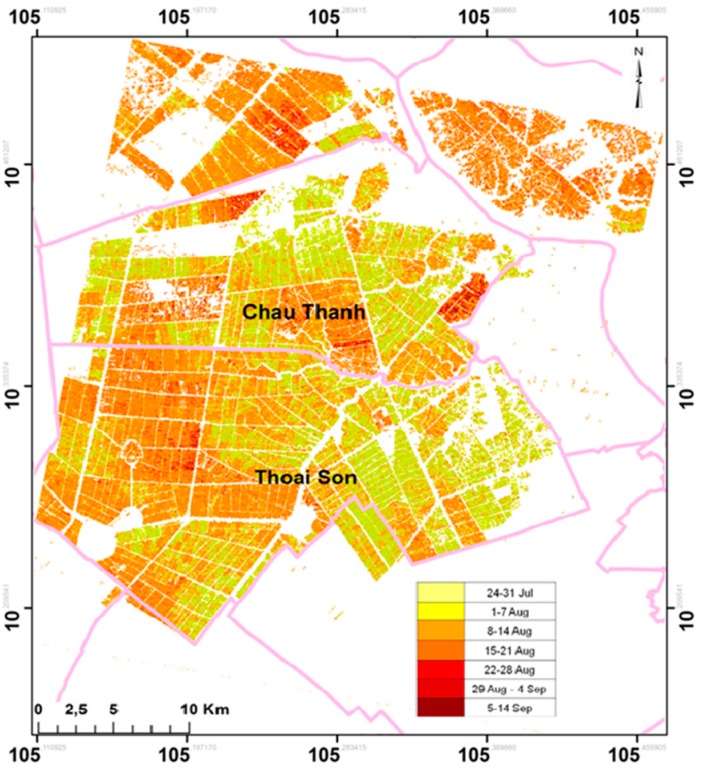
Sowing date map in Autumn-Winter crop in Chau Thanh and Thoai Son districts, An Giang Province.

Figure 12 shows the rice sowing dates map of the Autumn-Winter crop in Chau Thanh and Thoai Son communes. The result indicates clearly that the sowing dates span from the end of July to mid-September, although the common knowledge for Autumn–Winter crop sowing period in the region was restricted to Middle August. The result shows that at the Northern part of the study site, sowing dates tend to be later than Middle August, and at the Southern part, mixed earlier and later sowing dates are found.

The map in Figure 12 shows scatterered pixels within blocks of rice fields, due to the uncertainties in the sowing date determined by interpolation of the HH/VV curves. A post processing filter could be applied to the rice sowing dates map in Figure 12 to reduce these scattered pixels. However, in some cases, there are narrow fields inside blocks of fields with different sowing dates and varieties. If field boundaries are available, sowing date estimation could also be better estimated at the field basis. 

### 4.3. Long/Short Cycle Rice Mapping

**Figure 13:**
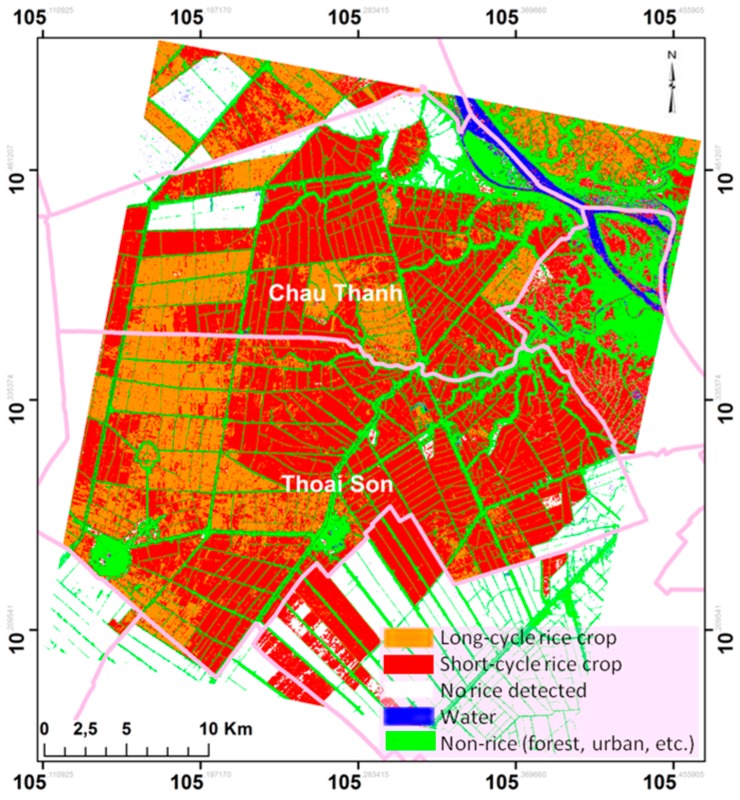
Rice varieties map using polarization ratio method. Orange: long-cycle rice crop (30.3%); red: short-cycle rice crop (69.7%); Blue: Water; Green: built areas and trees; White: no rice detected.

The long/short rice cycle map has been generated and shown in Figure 13. Short and long cycle rice fields are distributed in geographic clusters. Short-cycle rice is still dominant (69.7%), desite the tendancy to increase the production of long-cycle rice. It is noted that the proportion is different from the distribution of the sampled fields, which include 42% of short-cycle fields and 58% of long-cycle rice.

### 4.4. Accuracy Assessment

The results validation has been performed using independent in situ data collected for this purpose. For rice/non-rice detection, the rice mapping result is compared with 64 independent ‘check points’ localized using a GPS of rice/non rice from ground survey after the rice season. From the rice map generated using CSK, the pixels containing the ‘check points’ are extracted. The comparison in rice or non-rice is of 59 good identification among the 64 pixels (92%) (Cf. complementary information). However, the assessment includes also other error sources than the rice detection method. For example, the errors of the five erroneous points correspond to check points located less than 10 m from the roads, very likely due to localization error, given the accuracy of the GPS used (about 10 m). This source of error can be minimized in regions where field boundaries are available.

In order to complete the assessment, and for the purpose to meet the user requirements in rice statistics, we compare the mapping results using CSK with the published rice planted area in each of the 16 communes in the Thoai Son district. 

**Figure 14:**
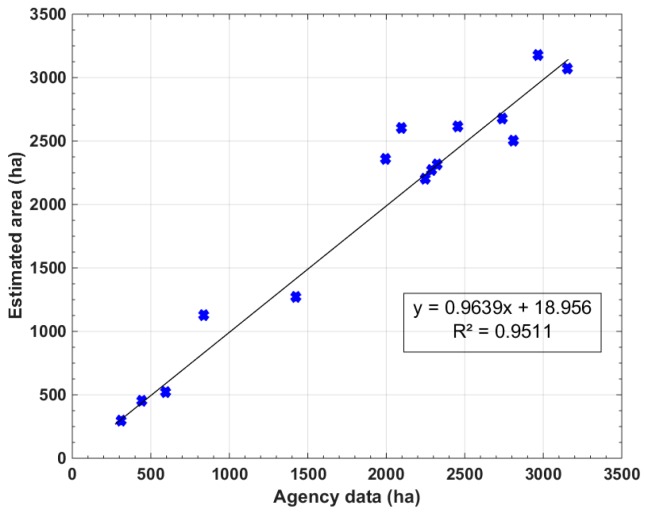
Estimated area in Autumn-Winter crop by CKS vs. agency data of 15 communes in Thoai Son districts, An Giang Province. Black line represents the linear regression between two datasets.

Table 4 shows a comparison between the the CSK-derived rice areas and data provided by the An Giang statistics office. Although both estimates have multiple sources of error, they provide good agreement in the overall comparison, the R² value is 0.95 (Figure 14).

For validation of the sowing dates and rice variety mapping, the 40 sampled fields are used as follows: we generate the results using 10 fields selected randomly among 40 fields in the training phase. The 30 other surveyed fields were considered as independent data for validation of sowing dates and rice variety maps, since these data have not been used for the training of the methods.

**Figure 15:**
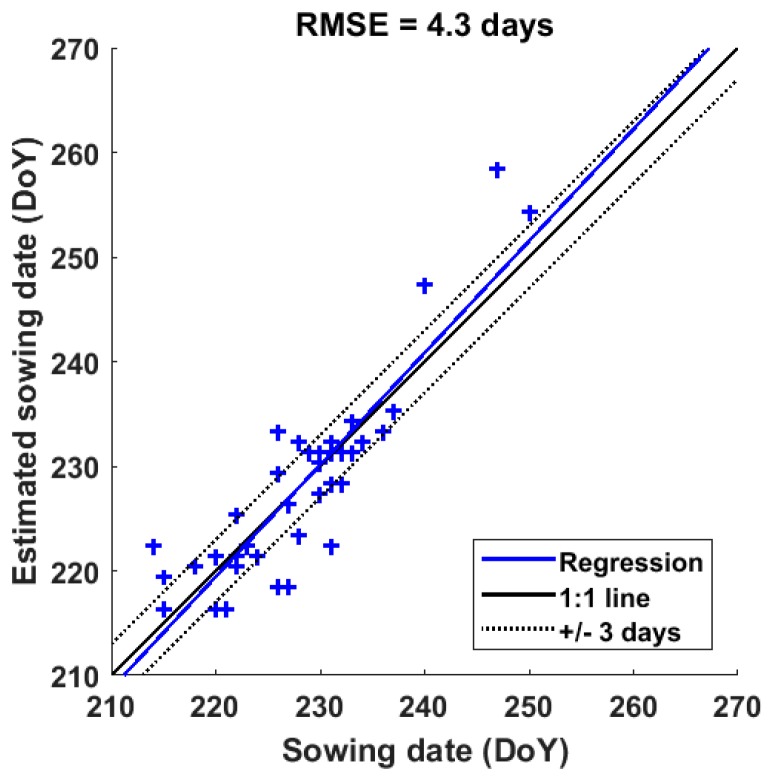
Retrieved sowing date in Autumn-Winter crop by CKS vs. ground data collection of 30 rice fields in Chau Thanh and Thoai Son districts, An Giang Province. Blue line represents the linear regression between two datasets.

The sowing date map derived from CSK data is compared with the sowing dates of 30 sampled fields showing a good agreement with a root mean square error of 4.3 days (Figure 15). 

Determining the sowing date of rice fields in this region with an error of ± 4.3 days is an asset for water management, field treatment and harvest planning. The current planning for these operations is based on a sowing date around mid-August, whereas the mapping results indicate large span of the sowing date (end of July to mid-September). However, for individual rice fields, an error of 4–5 days may not be sufficient for punctual management, and the contribution of the spatial distribution of sowing dates is more important at a regional scale, where water resources and available machinery need to be shared between communes and villages.

The comparison between rice variety mapping in this study and 28 sampled fields (two rice fields among 30 were without information of rice variety) has shown that only for one field of the long rice cycle variety was not correctly detected. The good identification score is therefore of 27/28 (96%). More important is the derived area distribution and the proportion of long and short cycle rice within a region. In the study area (in Figure 13), 30.3% of the area is planted with of long-cycle rice and 69.7% with short-cycle rice. Such proportion is expected to change annually.

The proportion of planted area with long-cycle high market value rice in a season against planted area with short-cycle lower market value rice is a major component of the economic income in this region. Having this information 1 month to 1 month and a half after the sowing period will be beneficial for market planning.

### 4.5. Discussions

In this section, the methods developed in this paper are discussed with respect to their application to other regions, and their generality concerning SAR data with different frequency and polarization. The use the HH/VV polarization ratio for rice field identification has been indicated since early 90s, when Le Toan et al. [14] has shown that the ratio is a better indicator for rice field mapping thanks to the vertical structure of the rice plants. The vertically polarized wave is more attenuated than the horizontally polarized wave, and for that reason the ratio of the HH and VV backscatter intensities is higher than that of most other land cover classes. This specific feature relies on the structure of rice plants, therefore will hold for rice fields at different regions. The higher wave attenuation in VV compared to HH is expected at X, but also at C and L bands, so that the methods can be used for C-band and L-band data. For example, existing studies using the HH/VV polarization ratio for rice mapping are mostly at C-band [6,7]. However, using Sentinel-1 data, the available polarizations for land are VV and VH. The differences between these polarizations do not arise from differential wave attenuation and need to be studied. 

For the detection of the sowing date, in past studies, methods based on optical data [39,40] are limited because of the frequent cloud cover, and methods based on SAR [24,35,41] relied on assumptions of flooded fields at the Start of Season, that do not apply universally at present, as mentioned previously. In this study, the sowing date is based on the particular polarization behavior of rice plants at X-band, with HH close to VV due to the particular structure of the rice plant at a specific phenological stage, which is the beginning of tillering, at about 20 days, where the leaves lose its vertical structure and the stem is still small. These specific features, the plant age of about 20 days at the beginning of tillering, the plant structure at the beginning of tillering, and the close values of HH and VV at X-band were found common for varieties in this study, and very different rice varieties in France [14]. Detecting this phenological stage and determining the sowing date could be generalisable, providing that a calibration of the method could be done to take into account the effects of cultural practices and SAR data observing parameters (frequency, incidence angle).

The algorithm to detect long versus short cycle rice is based on the higher backscatter of the long cycle rice at the reproductive stage (due to the higher value of plant height). As long as the long cycle rice continues to grow after the short cycle rice, this will hold for other regions, and other radar frequencies. To apply the methods in different regions, we will need to know the general information on the cultivation practices, crop calendar, rice variety and its duration. This will have an impact on the threshold values used in the mapping methods, the overall methodology will remain the same.

The achieved maps in this study include general LULC and rice growing areas sowing date map and long/short cycle rice map for Autumn-Winter rice season in 2013 in the CSK frame covering Chau Thanh and Thoai son districts, in the An Giang Province. For results validation, a standardized validation method (e.g., including sampling strategy and design, response design, etc., as proposed in [42]) should be applied. However, due to the time and budget constraints, we have used only the (64) independent GPS check points and the agency statistics published well after the rice season for rice/non rice mapping accuracy. For more statistically sound validation, a higher number of GPS check points is preferable in future experiments. For long/short cycle rice and sowing date, we have used the 40 surveyed fields, from which 10 random fields are used for training and 30 fields for comparison with the results. The maps can be used at local scale for planning of farm activities, for organizing labor at the right time. It also provides accurate information for the local government, planners and decision makers, to assess the rice grown area for each rice season, and to have early estimation of long and short cycle rice with different market values. However, for province and national scale mapping, the coverage of the X-band SAR (17 km) is a limitation. In this case, large coverage data with frequent repeat cycle such as Sentinel-1 will be more adapted. 

## 5. Conclusions

The aim of this study was to assess the use of SAR images for rice mapping, not only restricted to mapping of rice growing areas but extending to the retrieval of the sowing date, which is the information relevant to field management and, and the short and long rice varieties, which is important to market assessment. 

For this purpose, the study, using COSMO-SkyMed X-Band SAR data, relies on the analysis of the temporal variation of the SAR intensity as a function of short-cycle and long-cycle rice varieties, and the field sowing date, in the region of An Giang in the Mekong Delta. Firstly, a synthesis of rice crop characteristics and cultural practices in the region was given, based on the survey information. The differences between the temporal behavior of backscatter intensity HH, VV and HH/VV ratio of rice and other LULC classes in the region, and the differences between short-cycle and long-cycle varieties were analysed to derive indicators relevant to rice mapping and rice varieties discrimination, and relevant to the sowing date determination. The study has pointed out the following important backscatter temporal behaviors:

(1) As indicated by previous studies, the HH/VV ratio can be used for mapping of actual rice grown area after at least three satellite data acquisitions;
(2) The polarization ratio HH/VV is close to 0 dB at the tillering stage (about 20 days) when the plant loses its vertical structure. The result was exploited to retrieve the sowing date. For this purpose, data need to be acquired with relatively short repeat cycle for interpolation of the backscatter temporal curve at the 0 dB crossing line. For a 12 day repeat cycle for example, the sowing date could be retrieved 1 month after the beginning of the season;
(3) The differences in the value and the timing of the maximum of HH/VV ratio between the long-cycle and short-cycle rice after sowing are used to discriminate among the two varieties. Long-cycle rice ratio can reach maximum values of more than 10 dB at around 60 days after sowing, whereas it is less than 8 dB for the short-cycle rice at around 50 days after sowing. In addition, knowing the sowing dates and the rice varieties, the maturity phase and the harvest timing can be predicted;

Further works will consist of testing the methods at different rice growing regions, and assessing the methods using different SAR data. An X-band SAR satellite dedicated to a given region such as LOTUSat could be used for local to regional survey. With Sentinel-1 data available worldwide with free and open data access, it is expected that relevant information can be systematically retrieved, leading to effective large scale rice monitoring operations.

## Figures and Tables

**Table 1:** Main rice seasons in An Giang, Mekong River Delta, Vietnam.

Rice crop season	Sowing/Transpanting	Harvest
Winter-Spring	Nov-Dec	Mar-Apr
Summer-Autumn	Apr-May	Jul-Aug
Autumn-Winter	Jul-Sep	Oct-Dec
Rainy season	Jul-Sep	Nov-Jan

**Table 2:** Example of rice plant parameters measured at some sample rice fields.

	General Parameters			Plant Height (cm)						
Plot	Sowing D.	Harvest D.	Variety	20130830	20130904	20130913	20130926	20131006	20131017	20131028
1	20130816	20131110	50404	15.21	29.7	45.6	48.3	54.2	71.8	64.6
2	20130808	20131106	50404	18.0	33.0	43.0	54.24	58.8	68.4	65
3	20130811	20131107	50404	21.66	45.0	44.56	48.72	62.2	75.6	69.4

**Table 3:** Ground survey of the sowing date and harvest date (in yyyymmdd) of 40 rice field samples during the Autumn–Winter rice crop season 2013 in Chau Thanh and Thoai Son districts, An Giang province.

Plot	Sowing Date	Harvest Date	Plot	Sowing Date	Harvest Date
1	20130816	20131110	21	20130820	20131128
2	20130808	20131106	22	20130907	20131217
3	20130811	20131107	23	20130814	20131110
4	20130819	20131113	24	20130819	20131113
5	20130815	20131112	25	20130822	20131120
6	20130814	20131109	26	20130821	20131116
7	20130802	20131106	27	20130824	20131118
8	20130825	20131207	28	20130810	20131114
9	20130810	20131108	29	20130806	20131111
10	20130818	20131128	30	20130814	20131120
11	20130828	20131130	31	20130816	20131121
12	20130803	20131110	32	20130810	20131116
13	20130904	20131217	33	20130812	20131113
14	20130825	20131117	34	20130808	20131119
15	20130821	20131116	35	20130808	20131110
16	20130817	20131118	36	20130809	20131112
17	20130819	20131126	37	20130815	20131124
18	20130818	20131128	38	20130803	20131102
19	20130820	20131113	39	20130818	20131108
20	20130819	20131128	40	20130815	20131108

**Table 4:** Statistics of rice planted areas in 15 communes in Thoai Son district in Autumn-Winter 2013 rice season.

Commune Name	An Binh	Binh Thanh	Dinh My	Dinh Thanh	My Phu Dong	Phu Thuan	Thoai Giang	Nui Sap	Oc Eo	Phu Hoa	Vinh Khanh	Vinh Phu	Vinh Trach	Vinh Chanh	Vong Dong
Estimated area (ha)	2274	2313	3178	2613	2676	1131	2203	455	522	294	2500	3069	1269	2357	2601
Agency area (ha)	2287	2325	2965	2454	2740	835	2248	442	592	310	2810	3153	1424	1996	2096
Percentage error (%)	−0.55	−0.52	6.70	6.09	−2.39	26.19	−2.03	2.82	−13.5	−5.5	−12.39	−2.73	−12.19	15.31	19.42

## Supplementary Materials

The following are available online at http://www.mdpi.com/1424-8220/18/1/316/s1.
